# Effects of short‐ and long‐term use of propolis extracts on liver and kidney in rats

**DOI:** 10.1002/fsn3.4199

**Published:** 2024-05-13

**Authors:** Sibel Silici, Sevim Demiray, Aslı Okan, Sena Ertuğrul, Sahar Alizada, Züleyha Doğanyiğit

**Affiliations:** ^1^ Department of Agricultural Biotechnology, Faculty of Agriculture, Nutral Therapy Erciyes University Kayseri Turkey; ^2^ Department of Histology and Embryology, Faculty of Medicine Yozgat Bozok University Yozgat Turkey; ^3^ Gulhane Medical Faculty University of Health Sciences Ankara Turkey; ^4^ Cerrahpasa Medical Faculty Istanbul University‐Cerrahpasa Istanbul Turkey

**Keywords:** ethanol, hepatic, olive oil, propolis, renal

## Abstract

Propolis is widely used as a supplementary food product for its health benefits. The aim of this study was to determine the effects of commercial propolis extracts on the liver and kidney. Propolis extracts (250 mg/kgbw/day) were administered orally to adult male *Wistar albino* rats in solvents of ethanol, propylene glycol, water, and olive oil. Liver enzyme levels were determined biochemically in blood samples, and histopathological examinations were performed on the liver. Damage rate in both kidney tissue in the propolis‐ethanol extract group increased significantly compared with the other groups after 30 and 90 days of application (*p* < .05). According to the results, ethanol, used as a common solvent in propolis products, may adversely affect the liver in long‐term use. The data indicate that propolis‐olive oil extract may be an essential alternative due to its effective and reliable properties.

## Graphical abstract

In our study, we used the propolis derived from black poplar *(Populus nigra*). To assess long‐term effects of various propolis extracts, 80 male Wistar albino rats were divided into ten groups and received daily oral doses of 250 mg ethyl alcohol, propylene glycol, water, and olive oil‐based propolis extracts for 30 and 90 days. Liver enzyme levels (GGT, AST, ALT, ALP, and LDH) were measured in blood samples, and histopathological examinations were conducted on liver and kidney tissues.

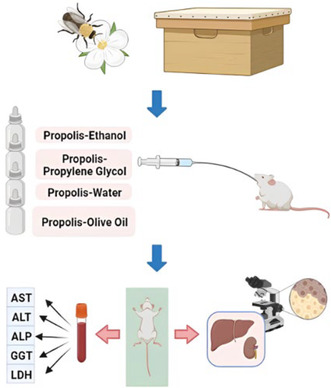

## 1 INTRODUCTION

Propolis is derived from the resin that honeybees (*Apis mellifera* L.) collect from plants (de Groot, 2013). It is used for beehive construction, repair, and disinfection (Pasupuleti et al., 2017). Propolis contains 50% resin and vegetable balms, 30% beeswax, 10% essential oils, 10% terpenes, 10% pollen, and other organic substances (Burdock, 1998). In recent years, due to the potential for the development of new medicines, propolis has been extensively studied. It has immunomodulatory, anti‐inflammatory, antioxidant, antibacterial, antiviral, antifungal, and antiparasitic bioactivity (Sforcin & Bankova, 2011). The extraction must take place with solvents, as raw propolis is not suitable for consumption (Pietta et al., 2002). Solvents in propolis extraction are divided into two groups organic (water and oil) and alcoholic (ethyl alcohol, propylene glycol, glycol, glycerol, etc.). The antimicrobial characteristics of the extract, extraction yield, and content of phenolic and flavonoid substances can be influenced by the propolis extraction method (Pobiega et al., 2019). Propolis extraction with alcohol is a simple and effective method (Silici & Baysa, 2020). The ethanolic extract of propolis (EEP) is a rich source of phenolic acids and flavonoids. Studies confirm that the extraction of propolis with ethanol has immunomodulatory, chemo‐preventive, and antitumor effects (Sforcin, 2007). Even though there are beneficial effects of EEP on health, it can also show toxic effects (Bonamigo et al., 2017).

Ethyl alcohol, which is a commonly used solvent in propolis extraction, is rapidly absorbed from the gastrointestinal tract upon oral ingestion and distributed throughout body fluids. The rate of distribution of ethyl alcohol in body fluids and tissues depends on the size, permeability, and blood flow of the tissue. Ethyl alcohol is mainly (90%) metabolized in the liver. The main pathway in the biotransformation of ethyl alcohol is its oxidation in the liver to acetaldehyde and hydrogen. In addition, ethanol and its metabolites are excreted in the urine, and its content in urine is higher than in blood and liver. Chronic alcohol administration reduces renal tubular reabsorption and renal function. Functional abnormalities of renal tubules may be associated with ethanol‐induced changes. For these reasons, we preferred both enzymatic and histological examination in liver tissue and histological examination in kidney.

Liver enzymes may be increased by prolonged use of propolis (D'Ercole, 2020). Chronic ethanol consumption harms hepatocytes through oxidative stress and lipid oxidation (Diehl, 2005). Like the liver, kidneys express cytochrome P450 2E1 enzyme, which catabolizes ethanol with free radical formation (Latchoumycandane et al., 2014). Therefore, ethanol catabolism can also damage the kidneys. Sudden loss of kidney function in patients with alcoholic hepatitis is an indicator of an increased risk of mortality (Altamirano et al., 2012). Although the daily consumption of propolis‐ethanol extract is low, this risk can pose liver and kidney health in long‐term use. Furthermore, the use of EEP in cancer and diabetes patients and children is limited. For this reason, researchers have been in search of more harmless extraction solvents (Sforcin, 2016).

In this study, we aimed to reveal the short‐ and long‐term effects of commonly used commercial propolis extracts on liver and kidney tissues in rats. Liver enzymes such as gamma‐glutamyl transpeptidase (GGT), aspartate aminotransferase (AST), alanine aminotransferase (ALT), alkaline phosphatase (ALP), and lactate dehydrogenase (LDH) and liver and kidney tissue histology were examined to determine these effects.

## 2 MATERIALS AND METHODS

### 2.1 Animals

Ten separate groups were randomly selected from a total of 80 adult male Wistar albino rats (8 weeks age, 240–280 g). For 30 and 90 days, oral gavage was used to give ethanol, olive oil, propylene glycol, and a water‐based propolis extract (each dose individually) (Eraslan et al., 2008). The rats were housed in cages, fed with standard commercial pellet food, and maintained in a regular laboratory environment with 12‐h light/dark cycles. Feed and water were given ad libitum. After the experiment, rats were given ketamine/xylazine anesthesia to minimize the pain of sacrifice while having blood samples extracted from the heart. Ethics committee approval was obtained from Erciyes University Animal Experiments Local Ethics Committee (Ethics Committee Approval No: 04.03.2020‐20/063) for the study. There were eight rats in each group.

### 2.2 Propolis

The propolis sample used in the research was prepared by Erciyes University Technopark (Nutral Therapy). The botanical origin of the propolis used in this study is black poplar (*Populus nigra* L.) and its geographical origin is Kayseri (Central Anatolia, Turkey). For extraction, raw propolis samples were ground and homogenized. Twenty‐five grams of propolis powder was extracted from 100 mL of ethanol–water (70:30), distilled water, olive oil, and propylene glycol (PG), respectively. The ratio of the solid material to the solvents was 4:10. Ultrasound extraction was conducted according to the method of Netíková et al. (2013) with some modifications. Briefly, the solid–solvent mixtures were shaken for 24 h at 20°C using a shaking water bath. Then ultrasound‐assisted (UA) and conventional (C) extracts were centrifuged at 4427× *g* for 10 min. The extracts were then filtered through a Whatman No. 1 filter paper and stored at +4°C.

### 2.3 Study design

Group 1: Control I (30 days, oral gavage with physiological saline) (*n* = 8).
Group 2: Control II (90 days, oral gavage with physiological saline) (*n* = 8).
Group 3: Water extract of propolis (WEP I; 250 mg/kgbw/day), 30 days, oral gavage (*n* = 8).
Group 4: Water extract of propolis (WEP II; 250 mg/kgbw/day) 90 days, oral gavage (*n* = 8).
Group 5: Ethanol extract of propolis EEPI; (250 mg/kgbw/day) 30 days, oral gavage (*n* = 8).
Group 6: Ethanol extract of propolis (EEPII; 250 mg/kgbw/day) 90 days, oral gavage (*n* = 8).
Group 7: Propylene glycol extract of propolis (PGEPI; 250 mg/kgbw/day) 30 days, oral gavage (*n* = 8).
Group 8: Propylene glycol extract of propolis (PGEPII; 250 mg/kgbw/day) 90 days, oral gavage (*n* = 8).
Group 9: Olive oil extract of propolis (OOEPI; 250 mg/kgbw/day) 30 days, oral gavage (*n* = 8).
Group 10: Olive oil extract of propolis (OOEPII; 250 mg/kgbw/day) 90 days, oral gavage (*n* = 8).

### 2.4 Analysis of blood samples

Blood samples were collected from the animals only by the end of the 30 and 90 days of the study. The animals were fasted for 6 h before blood samples were taken. General anesthesia was not given before the samples were taken since there was a chance that the biochemical parameters may alter. Just prior to taking blood samples, the animals were kept under a light ether anesthetic. Blood samples were then slowly taken from each animal into tubes both with and without anticoagulants using a cannula placed in the heart. Blood was taken from every eight animals in each group. Blood samples were collected into both types of tubes, and analyses for the parameters under investigation were done on the same day. Utilizing a Konelab 60i model auto‐analyzer and a kit from the same manufacturer, serum biochemical parameters including GGT, LDH, AST, ALT, and ALP were measured using Johnson & Johnson Label kits (Eraslan et al., 2009).

### 2.5 Histological analysis

The tissues collected at the end of the experiment were fixed with 10% formalin for 1 week. The tissues were then dehydrated through 50%, 70%, 80%, 96%, and 100% ethanol, cleared with xylene, and embedded in paraffin wax. Sections were cut at 5 μm and stained with Harris Hematoxylin Eosin, mounted with Entellan (Merck, Darmstadt, Germany) to slides. Olympus, Tokyo, Japan's BX53 light microscope was used to analyze the preparations (Doǧanyiǧit et al., 2020). In the kidney sections, enlargement of the tubular lumen, glomerular degeneration, vacuolization, bleeding, and shedding of cells in the tubule were examined and tubular debris was assessed. Dilated blood vessels, inflammatory cell infiltration, and damaged hepatocytes with pycnotic nuclei were evaluated in the liver sections. Histopathological results in each category were scored as “0 = absent, 1 = mild, 2 = moderate, and 3 = severe” (Doǧanyiǧit et al., 2020; Inandiklioglu et al., 2021). Quantification was performed by two investigators blindly.

### 2.6 Statistical analysis

Biochemical data were presented as mean ± standard error of mean (SEM). Statistical Package for Social Sciences (SPSS) version 22.0 for Windows (SPSS Inc., Chicago, IL) was used to compare data across groups using one‐way analysis of variance (ANOVA) and post hoc Tukey honestly significant difference (HSD) tests were conducted. Histopathological score data expressed as ±SEM (standard error of the mean). Two‐way ANOVA (analysis of variance), Sidak's multiple comparison test (to examine the difference between 30 and 90 days of application) and TUKEY's multiple comparison test (to compare 30‐day and 90‐day treatment groups within themselves) were applied.

## 3 RESULTS

### 3.1 Histological analysis in kidney tissue of experimental groups

**FIGURE 1:**
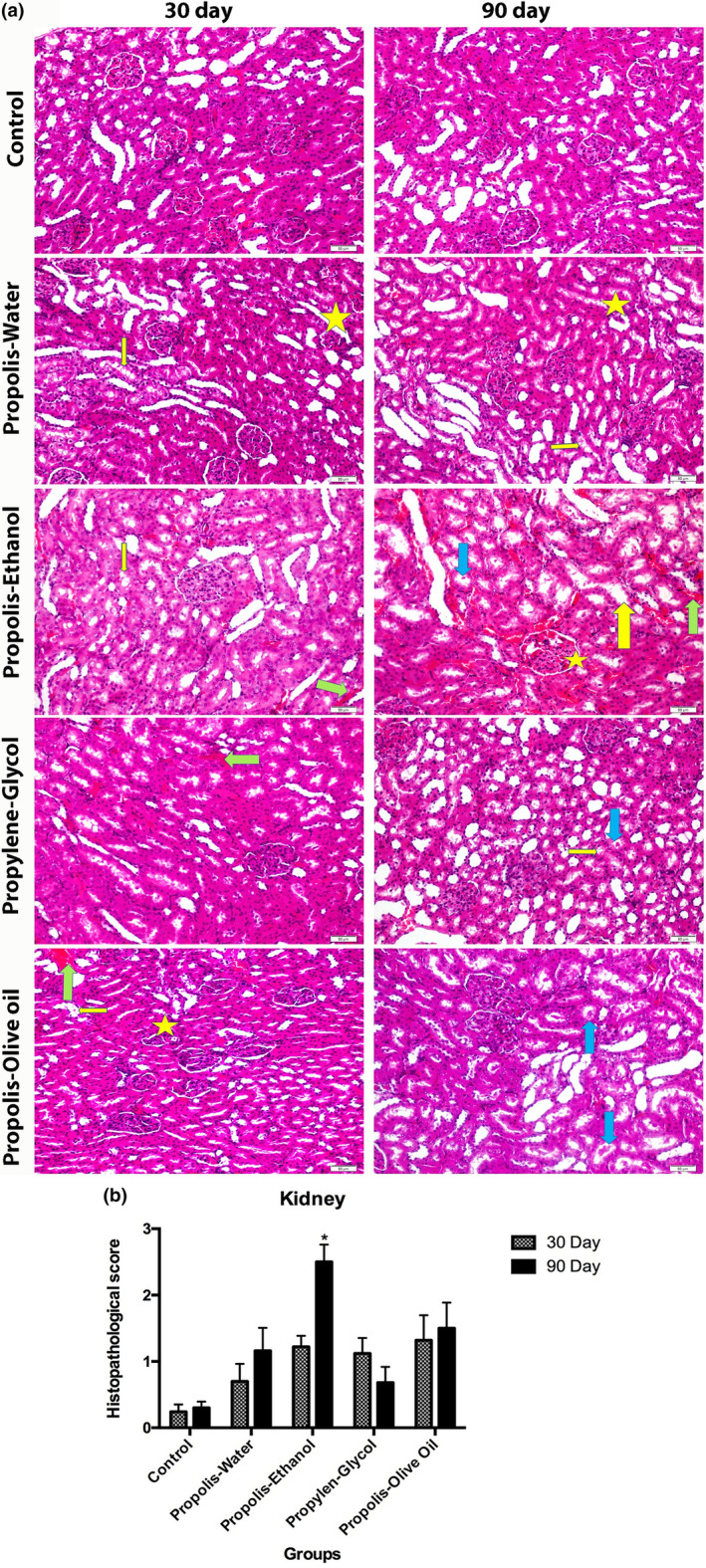
Histopathological analysis of the kidney. (a) Hematoxylin eosin staining images of rat kidney tissues belonging to the experimental groups (star: glomerular degeneration, green arrow: bleeding, yellow arrow: enlargement of the tubular lumen, thin yellow arrow: vacuolization, blue arrow: tubular debris). (b) *p < 0.0001 represents statistically significant difference.

The effects of ethanol‐, propylene glycol‐, water‐, and olive oil‐based extracts of propolis on kidney tissue were histologically evaluated for 30 and 90 days. As shown in Figure 1a, kidney images of control groups exhibited normal histological structure. Especially in the propolis‐ethanol group, vacuolization, and bleeding were observed in 30 days of application. In the 90‐day practice, the enlargement of the tubular lumen, bleeding, and the residual materials pouring into the tubule were concentrated. When we compare the 90‐day treatment groups, propolis‐ethanol group's damage rate analyzed in these groups significantly increased compared to the other groups (*p < .05). As we examined the difference between the amount of damage after 30 and 90 days of application in the groups, only the propolis‐ethanol group's damage rate at the end of the 90 days significantly increased compared with the 30 days (*p < .05) (Figure 1b).

### 3.2 Histological analysis in liver tissue of experimental groups

**FIGURE 2:**
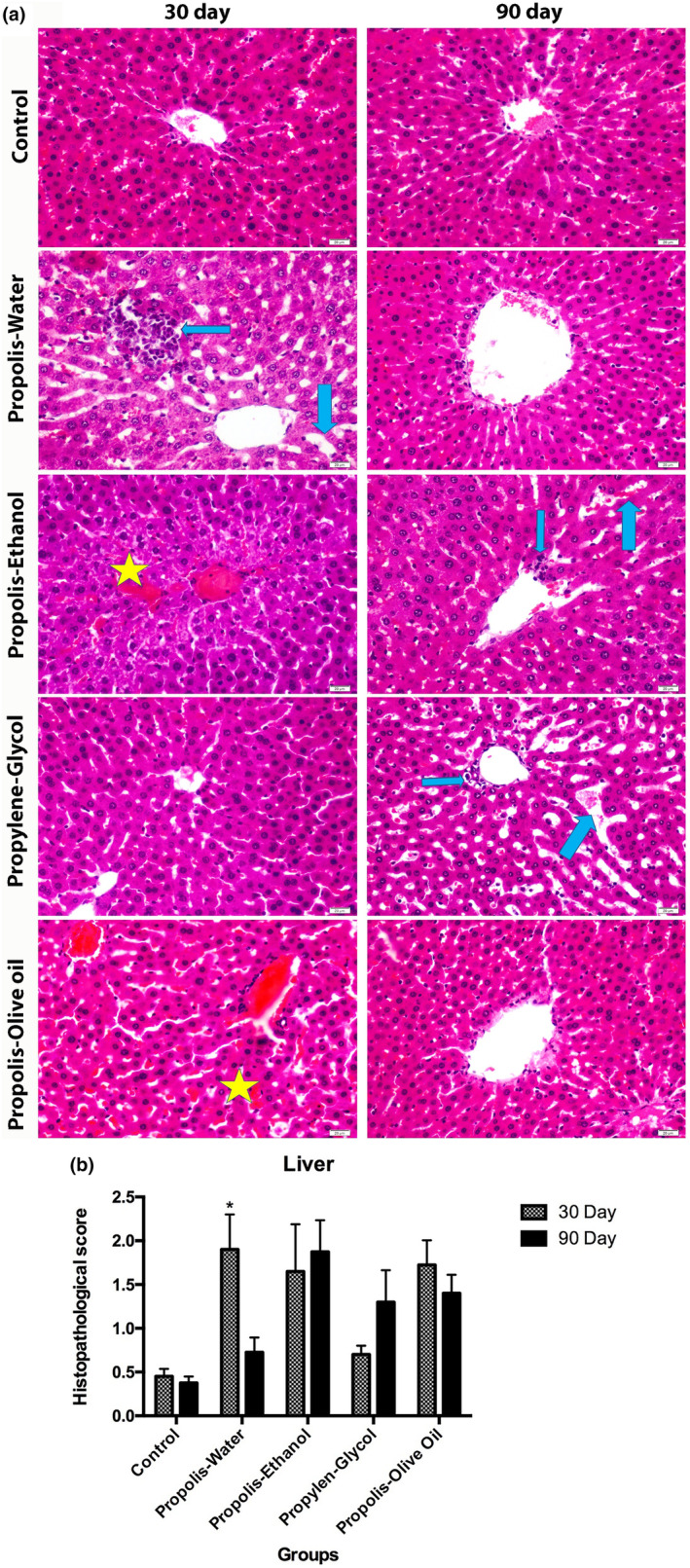
Histopathological analysis of the liver. (a) Hematoxylin eosin staining images of rat liver tissues belonging to the experimental groups (thick blue arrow: sinusoidal dilatation; thin blue arrow: inflammatory cell infiltration and asterisk: bleeding). (b) Histopathological score data shown in the histogram graph. Expressed as ±SEM (standard error of the mean). Two‐way analysis of variance (ANOVA) and (to examine the difference between 30 and 90 days of application) and TUKEY's multiple comparison test (to compare 30‐day and 90‐day application groups) were applied (*p < 0.0001 represents statistically significant difference).

The effects of ethanol‐, propylene glycol‐, water‐, and olive oil‐based extracts of propolis on liver in rats were histologically evaluated during 30 and 90 days. As shown in Figure 2a, liver images belonging to the control groups exhibited normal histological structure. Inflammatory cell infiltration and dilated blood vessels increased in liver sections of rats treated with propolis‐water especially at the end of 30 days. The incidence of tissue damage decreased in the groups that were applied propolis‐water for 90 days. As the propylene‐glycol group was applied for 90 days, inflammatory cells increased, and sinusoidal dilatation was increased when compared with the group applied for 30 days (*p < .05). Bleeding was observed in the 30‐day application in the propolis‐ethanol group. In 90 days of application, bleeding decreased but inflammatory cells increased, and sinusoidal dilatation increased (*p < .05). When the other groups were compared in days, the rate of damage in the short‐ and long‐term applications was found to be similar (*p < .05) (Figure 2b).

When we examine the difference between the amount of damage after 30 and 90 days of application in the groups, only the WEP group damage rate after 90 days was significantly lower than at the end of 30 days (*p < .05).

### 3.3 Biochemical findings

**FIGURE 3:**
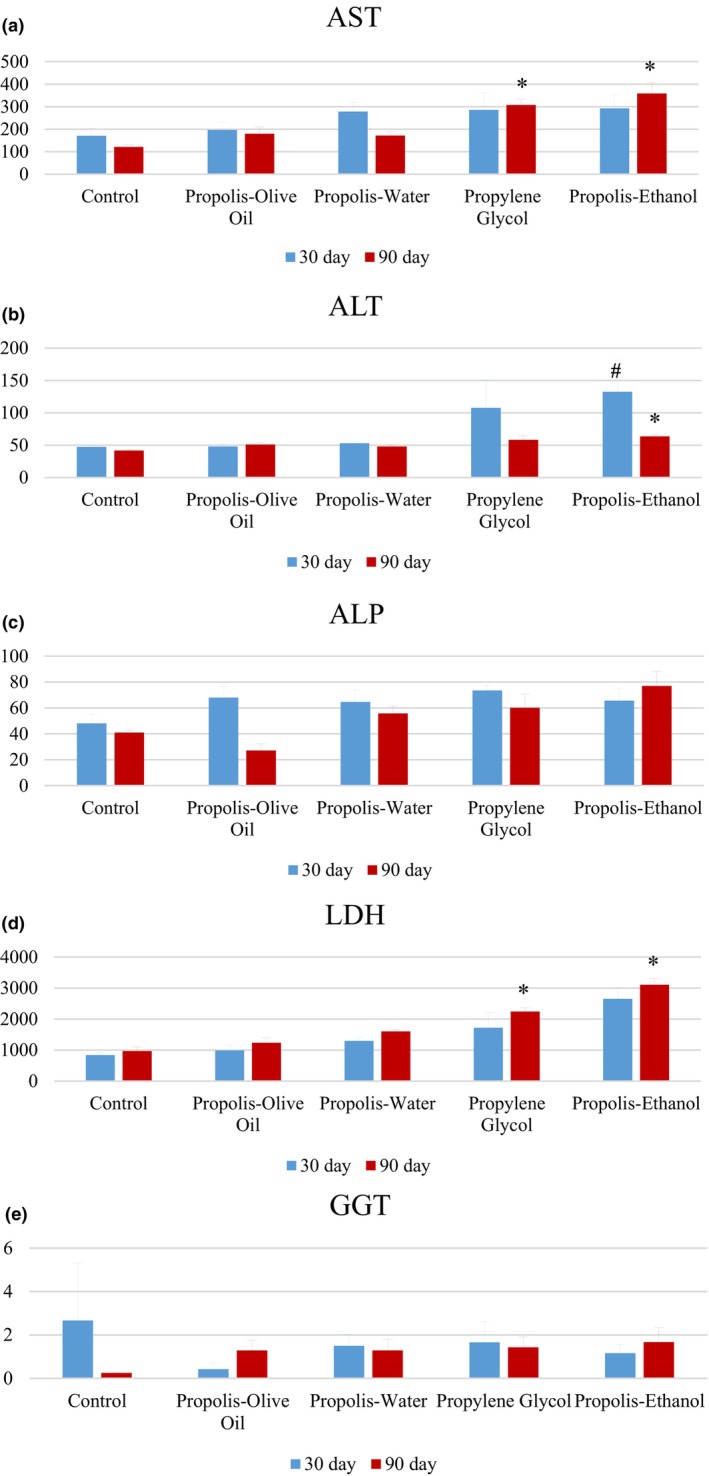
Biochemical analysis of liver. The effects of olive oil, water, propylene glycol and ethanol extract of propolis on aspartate transaminase (a), alanine transaminase (b), alkaline phosphatase (c), lactate dehydrogenase (d), and glutamyl transpeptidase (e) levels in blood samples were examined. Biochemical enzyme values shown in histogram graph. Expressed as ±SEM (standard error of the mean). Two‐way analysis of variance (ANOVA) (to examine the difference between 30 and 90 days of application) and TUKEY's multiple comparison test (to compare 30‐day and 90‐day treatment groups) were applied (ALP, alkaline phosphatase; ALT, alanine aminotransferase; AST, aspartate aminotransferase; GGT, gamma‐glutamyl transpeptidase; LDH, lactate dehydrogenase; ^#^ *p* < .05 represents that it is different from the control group in the 30‐day application groups, *p < .05 represents that it is different from the control group in the 90‐day application groups).

The effect of ethanol‐, propylene glycol‐, water‐, and olive oil‐based propolis application on the levels of liver enzymes AST, ALT, ALP, LDH, and GGT in blood samples was evaluated. At the end of 1 month application, AST levels increased in EEP, PGEP, and WEP groups compared with the control group, but this change was close to the control group in the OOEP group (Figure 3a). In ALT results, the EEP and PGEP groups were higher than the control (Figure 3b). As the LDH levels in blood were evaluated, the EEP group significantly increased (^#^ *p* < .05) compared with the control group (Figure 3d).

AST results showed a significant increase in ethanol and propylene groups after 3 months of application (*p < .05). In ALT results, the ethanol group significantly increased compared with control and other groups (*p < .05). As the LDH results were examined, the increase in the EEP and PGEP groups was significant compared with the other groups (*p < .05).

When all results are evaluated, enzyme activities in ethanol groups significantly increased (*p < .05), followed by PGEP and WEP groups, respectively, in the 1‐month and 3‐month applications. Hematological findings in rats given different propolis extracts for 90 days, AST, ALP, ALT, and LDH values were significantly different from each other.

## 4 DISCUSSION

In our study, we evaluated the histological changes, damage rates, and plasma levels of ALT, AST, ALP, LDH, and GGT enzymes to demonstrate the effect of solvents used in propolis extraction on liver and kidney tissues. We analyzed the differences in the control, OOEP, WEP, EEP, and PGEP groups through oral gavage administration of extracts.

Hepatoprotective and nephroprotective properties have been demonstrated for the active component of poplar‐type propolis, caffeine acid phenethyl ester (CAPE). A 15‐day study on rats showed that chestnut propolis has a protective effect against the oxidative damage caused by alcohol (Kolankaya et al., 2002). Another study demonstrated that CAPE protects from necrosis, lipid peroxidation, p65 activation, and abnormal cell proliferation (Macias‐Perez et al., 2013). Additionally, it was observed that preapplication with a single dosage of CAPE reduced the incidence of hepatic tumors caused by diethylnitrosamine (DEN) by 43% and that CAPE protects against aflatoxin B1‐induced hepatotoxicity through the control of free radical generation (Akcay et al., 1994; Beltrán‐Ramírez et al., 2012).

Furthermore, it was discovered that CAPE reduced systemic inflammation brought on by LPS and galactosamine in rats, which reduced damage to hepatic and neural cells (Korish & Arafa, 2011). Additionally, hepatic steatosis brought on by a high‐fat diet in a mouse model was seen to be improved by CAPE treatment. This improvement was associated with reductions in c‐N‐terminal kinase and nuclear factor kappa‐B (NF‐kB) activation as well as decreased expression of cyclooxygenase (COX) 22 (Bezerra et al., 2012). In a study utilizing electron microscopy, it was demonstrated that CAPE protects hepatocytes against ultrastructural changes induced by cholesterol (Esrefoglu et al., 2012). The antioxidant activity of CAPE has been shown to reduce oxidative damage in the liver, as well as mitigate cytokine damage and necroinflammation in rats with biliary bleeding. In addition, preadministration of CAPE significantly prevented tamoxifen‐induced liver toxicity in rats, attributed to the attenuation of antioxidant lipid peroxidation and the restoration of enzyme activity (Albukhari et al., 2009). While CAPE has demonstrated hepatoprotective and nephroprotective effects, its long‐term benefits rely on appropriate pairing with the right extract.

The protective effects of EEP on alcohol‐induced fatty liver in rats were investigated, and 4‐week EEP treatment showed a restoration in liver enzyme levels (Ye et al., 2019). The oral administration of EEP for a duration of 7 days has been observed to upregulate the expression of genes crucial for the antioxidant pathway in the livers of mice (Hotta et al., 2020). These findings suggest that EEP may serve as a potential therapeutic agent to prevent diseases associated with oxidative stress. We investigated the effects of an ethanol extract of propolis on the liver over 30 and 90 days of application. The amount of damage to liver tissue significantly increased in the EEP group. Moreover, we observed elevated levels of liver enzymes AST, ALT, and LDH as a result of EEP application. Therefore, we postulate that prolonged EEP usage can damage the liver tissue, whereas its short‐term application exhibits liver protective effect.

EEP affects the kidney as well as the liver tissue. Susanto et al. (2018) investigated the nephroprotective effect of propolis (50 mg/kg) on interstitial fibrosis, systolic blood pressure, and body weight in mice with unilateral ureteral obstruction. As a result, propolis increased body weight by decreasing interstitial fibrosis and systolic blood pressure. Due to its ability to absorb and concentrate hazardous substances along with its high blood flow, the kidney is the target of toxic compounds. Azab et al. (2014) assessed the effectiveness of various natural compounds (curcumin, rosemary, and propolis) against gentamicin‐induced nephrotoxicity and histological and biochemical alterations in mice. In addition to greatly reducing the raised blood levels of urea, creatinine, and uric acid, co‐administration of curcumin, rosemary, and propolis significantly contributed to the detrimental structural alterations in the kidney brought on by gentamicin. In another study, Osman and Hafez Tantaway (2013) investigated propolis's total phenol and flavonoid content, in vitro antioxidant activity, and potential anti‐nephrotoxic properties in rabbits. Utilizing biochemical indicators (measured of blood urea and creatinine) and histological kidney alterations, propolis' protective effects on gentamicin‐induced nephrotoxicity in rabbits were assessed. In conclusion, gentamicin's histopathological and biochemical side effects were dramatically reduced after oral administration of the propolis. The kidney is a crucial organ that is important in the detoxification and elimination of xenobiotics. Therefore, it is crucial to study natural kidney‐protecting substances. Aldamash et al. (2016) tested the renoprotective effects of propolis against gentamicin‐induced renal toxicity in mice. With the administration of gentamicin, collagen, and reticular fibers were detected by histochemistry, and immunohistochemistry revealed an increase in kidney injury (Kim‐1 gene expression), oxidative stress (MDA gene expression), and apoptosis (caspase‐3 gene expression). Propolis and gentamicin co‐administration results in a significant reduction in BUN levels, a reduction in tubular damage, the appearance of healthy glomeruli with normal cellularity, a reduction in collagen and reticular fiber deposition, and a reduction in apoptosis, kidney damage, and oxidative stress. These results demonstrate the kidney‐protective role of propolis against gentamicin‐induced toxicity. Inflammation and oxidative stress both play pathogenic roles in chronic kidney disease (CKD). In experimental nephropathies, anti‐inflammatories and antioxidants significantly protect the kidneys. Red propolis from Brazil was used to create a kidney ablation model. When propolis was given to rats with significant proteinuria and hypertension 30 days after surgery, significant declines in glomerulosclerosis, proteinuria, serum creatinine retention, glomerulosclerosis, renal macrophage infiltration, and oxidative stress were observed 90 days later (Teles et al., 2015). In conclusion, propolis treatment reduced hypertension and structural kidney damage in the tested model. Baykara et al. (2015) showed that creatine urea, MDA, GSH, SOD, and GSH‐Px levels increased in rats resulting from the administration of contrast agent (Diatrizoate), and propolis normalized these values as effectively as N‐acetyl cysteine and showed nephroprotective activity.

Chronic kidney disease can develop as a result of damaged tubular epithelial cells secreting pro‐inflammatory cytokines and profibrotic substances (Geng et al., 2012; Liu et al., 2018; van Kooten et al., 1999; Yard et al., 1992). Accordingly, chronic use of EEP may damage the renal tubules, causing serious kidney diseases such as CKD.

Another solvent tested in propolis extracts is propylene glycol in this study. We investigated the effects of PGEP on the liver and kidney. In our study, the increase in exposure time to PGEP significantly increased the damage rate in both kidney and liver tissue. In addition, the rate of kidney and liver damage at the end of 30 days in the propylene glycol group increased compared to the control and olive oil groups. In this group, the increase in ALT, AST, and LDH enzyme levels at the end of 90 days is indicative of liver damage. A study investigating the pharmacokinetics of propylene glycol showed that kidney's ability to excrete propylene glycol declines at large concentrations (Speth et al., 1987). Accordingly, 3‐month PGEP was more harmful compared with 1 month may be related to the decrease in kidney excretion. Propylene glycol is generally considered to be non‐toxic. However, excessive propylene glycol use causes metabolic acidosis and acute kidney damage (Bruns et al., 1982). On the other hand, PGEP has been found to suppress bacterial growth and biofilm production (Meto et al., 2020). Therefore, propolis is envisioned as a valuable source of natural compounds for the development of new therapeutic strategies against biofilm‐associated infections. These studies indicate that although PGEP has a beneficial effect, excessive and prolonged use can cause toxicity.

According to the study on the effects of WEP, it was observed that WEP has protective effects against influenza A virus and ultraviolet rays (Saito et al., 2015; Takemura et al., 2012). As compared to EEP, WEP has higher antioxidant activity and is effective for most harmful microorganisms. Using microkernel experiments, the use of WEP has been shown to be safe (Rocha et al., 2013). However, in our study, the amount of damage to the liver tissue significantly increased after the long‐term WEP application. Histochemical examination revealed that enlarged blood vessels and inflammatory cell infiltration increased in the liver at the end of 30 days. Similarly, another study found that WEP can suppress mitochondrial respiration in the heart (Majiene et al., 2006). Therefore, we assume that WEP can damage liver and heart tissue. Although water is a natural solvent, the use of chemicals to increase water solubility can pose a risk for WEP.

Efficacy of different propolis extracts was investigated in the literature. Propolis extracts using oil, ethanol, and propylene glycol all exhibited antibacterial and antifungal activities. However, the antimicrobial effect of glycerol solution was only temporary, lasting for a few days (Tosi et al., 1996). Researchers investigated the flavonoid content of crude propolis produced by Trigona bees in Indonesia (Sulawesi region) by extracting it with various solvents (water, ethanol, propylene glycol, olive oil, and coconut oil). They found that using oil as a solvent for propolis extraction resulted in comparable flavonoid content to that of the ethanol extract. Both olive oil and coconut oil were identified as suitable solvents for propolis extraction (Pujirahayu et al., 2014). In a study comparing various antioxidants, OOEP at 0.01% levels exhibited superior antioxidant activity than butylated hydroxyanisole and butylated hydroxytoluene. The highest antioxidant activity was observed at 0.08% concentration. The researchers concluded that antioxidant activity of propolis increased with higher concentrations and suggested that olive oil extract could serve as a natural antioxidant source (Özcan, 2000). The properties of the poplar‐type OOEP were investigated, focusing on its antioxidant, antiradical, and antipyretic effects, along with its total phenolic content. Increasing propolis concentration was found to enhance total phenolic content, antioxidant, and antiradical activity. Moreover, the olive oil extract of propolis demonstrated antipyretic effects in rats with yeast‐induced hyperthermia. These findings suggest that OOEP could serve as a healthier alternative to alcoholic extracts, displaying beneficial antioxidant, antiradical, and antipyretic effects (Silici & Baysa, 2020).

## 5 CONCLUSION

The biological effects of propolis extractions in long‐term use have not been adequately studied. Therefore, we examined effects of ethanol, propylene glycol, olive oil, and water extracts of propolis on kidney and liver tissues in short and long‐term use. Our study did not reveal any significant difference in the amount of damage to kidney and liver tissues in the propolis‐olive oil group compared with the control group. We postulate that olive oil‐based propolis extract could serve as a healthier substitute for alcoholic extracts. Moreover, the use of olive oil extract was thought to be safer than ethanol and propylene glycol extracts. In our biochemical analysis, a significant increase in AST, ALT, and LDH enzymes was interpreted as a result of ethanol extract use. Damage to the liver and kidney tissues of male adult *Wistar rats* may be associated with prolonged use of ethanol extract of propolis. As a result, attention should be paid to the dose and duration of use as well as the solvent used in the extraction of propolis to protect and support health.

## AUTHOR CONTRIBUTIONS

**Sibel Silici:** Conceptualization (lead); data curation (equal); formal analysis (equal); funding acquisition (lead); investigation (lead); methodology (equal); project administration (lead); resources (equal); supervision (equal); validation (equal); visualization (equal); writing – original draft (equal); writing – review and editing (equal). **Sevim Demiray:** Conceptualization (equal); data curation (equal); formal analysis (equal); funding acquisition (equal); methodology (equal); resources (equal); validation (equal); visualization (equal); writing – original draft (equal); writing – review and editing (equal). **Aslı Okan:** Data curation (equal); investigation (equal); methodology (equal); resources (equal); validation (equal); writing – original draft (equal); writing – review and editing (equal). **Sena Ertuğrul:** Data curation (equal); investigation (equal); methodology (equal); resources (equal); validation (equal); writing – original draft (equal); writing – review and editing (equal). **Sahar Alizada:** Conceptualization (equal); data curation (equal); methodology (equal); resources (equal); validation (equal); writing – original draft (equal); writing – review and editing (equal). **Züleyha Doğanyiğit:** Conceptualization (equal); data curation (equal); formal analysis (equal); funding acquisition (equal); investigation (equal); methodology (equal); project administration (equal); resources (equal); supervision (equal); validation (equal); visualization (equal); writing – original draft (equal); writing – review and editing (equal).

## FUNDING INFORMATION

This research was supported by the Erciyes University BAP (Scientific Research Projects) Unit (grant no. FBA 2020‐10528).

## CONFLICT OF INTEREST STATEMENT

There is no conflict of interest among the authors.

## ETHICS STATEMENT

Ethics committee approval was obtained from Erciyes University Animal Experiments Local Ethics Committee (Ethics Committee Approval No: 04.03.2020‐20/063) for the study.

## Data Availability

The data for the study can be made available from the corresponding author upon request.

## References

[fsn34199-bib-0001] Akcay, M. N. , Polat, M. , Cadirci, M. , & Gencer, B. (1994). Tumor‐induced ileo‐ileal invagination in adults. The American Surgeon, 60(12), 980–981. 7992979

[fsn34199-bib-0002] Albukhari, A. A. , Gashlan, H. M. , El‐Beshbishy, H. A. , Nagy, A. A. , & Abdel‐Naim, A. B. (2009). Caffeic acid phenethyl ester protects against tamoxifen‐induced hepatotoxicity in rats. Food and Chemical Toxicology, 47(7), 1689–1695. 19394397 10.1016/j.fct.2009.04.021

[fsn34199-bib-0003] Aldamash, B. A. , El Nagar, D. M. , & Elfakkilbrahim, K. (2016). Renoprotective effects of propolis on gentamicin‐induced acute renal toxicity in swis albino mice. Nefrología, 36(6), 643–652. 27575929 10.1016/j.nefro.2016.06.004

[fsn34199-bib-0004] Altamirano, J. , Fagundes, C. , Dominguez, M. , García, E. , Michelena, J. , Cárdenas, A. , Guevara, M. , Pereira, G. , Torres‐Vigil, K. , Arroyo, V. , Caballería, J. , Ginès, P. , & Bataller, R. (2012). Acute kidney injury is an early predictor of mortality for patients with alcoholic hepatitis. Clinical Gastroenterology and Hepatology, 10(1), 65–71.e3. 21946124 10.1016/j.cgh.2011.09.011

[fsn34199-bib-0005] Azab, A. E. , Fetouh, F. A. , & Albasha, M. O. (2014). Nephro‐protective effects of curcumin, rosemary and propolis against gentamicin induced toxicity in Guinea pigs: Morphological and biochemical study. American Journal of Clinical and Experimental Medicine, 2(2), 28–35.

[fsn34199-bib-0006] Baykara, M. , Silici, S. , Özçelik, M. , Güler, O. , Erdoğan, N. , & Bilgen, M. (2015). In vivo nephroprotective efficacy of propolis against contrast‐induced nephropathy. Diagnostic and Interventional Radiology, 21(4), 317–321. 26027766 10.5152/dir.2015.14075 PMC4498426

[fsn34199-bib-0007] Beltrán‐Ramírez, O. , Pérez, R. M. , Sierra‐Santoyo, A. , & Villa‐Treviño, S. (2012). Cancer prevention mediated by caffeic acid phenethyl ester involves cyp2b1/2 modulation in hepatocarcinogenesis. Toxicologic Pathology, 40(3), 466–472. 22291063 10.1177/0192623311431947

[fsn34199-bib-0008] Bezerra, R. M. N. , Veiga, L. F. , Caetano, A. C. , Rosalen, P. L. , Amaral, M. E. C. , Palanch, A. C. , & de Alencar, S. M. (2012). Caffeic acid phenethyl ester reduces the activation of the nuclear factor κB pathway by high‐fat diet‐induced obesity in mice. Metabolism, 61(11), 1606–1614. 22575582 10.1016/j.metabol.2012.04.006

[fsn34199-bib-0009] Bonamigo, T. , Campos, J. F. , Alfredo, T. M. , Balestieri, J. B. , Cardoso, C. A. , Paredes‐Gamero, E. J. , de Picoli, S. K. , & Dos Santos, E. L. (2017). Antioxidant, cytotoxic, and toxic activities of Propolis from two native bees in Brazil: *Scaptotrigona depilis* and *Melipona quadrifasciata anthidioides* . Oxidative Medicine and Cellular Longevity, 2017, 1038153. 28377794 10.1155/2017/1038153 PMC5362732

[fsn34199-bib-0010] Bruns, D. E. , Herold, D. A. , Rodeheaver, G. T. , & Edlich, R. F. (1982). Polyethylene glycol intoxication in burn patients. Burns, Including Thermal Injury, 9(1), 49–52. 7172076 10.1016/0305-4179(82)90136-x

[fsn34199-bib-0011] Burdock, G. A. (1998). Review of the biological properties and toxicity of bee propolis (propolis). Food and Chemical Toxicology, 36(4), 347–363. 9651052 10.1016/s0278-6915(97)00145-2

[fsn34199-bib-0012] de Groot, A. C. (2013). Propolis: A review of properties, applications, chemical composition, contact allergy, and other adverse effects. Dermatitis, 24(6), 263–282. 24201459 10.1097/DER.0000000000000011

[fsn34199-bib-0013] D'Ercole, M. C. (2020). Prolonged use of propolis can increase liver enzymes. Journal of Gastrointestinal and Liver Diseases, 29(3), 468–469. 32830823 10.15403/jgld-2582

[fsn34199-bib-0014] Diehl, A. M. (2005). Recent events in alcoholic liver disease V. effects of ethanol on liver regeneration. American Journal of Physiology. Gastrointestinal and Liver Physiology, 288(1), G1–G6. 15591584 10.1152/ajpgi.00376.2004

[fsn34199-bib-0015] Doǧanyiǧit, Z. , Kaymak, E. , & Silici, S. (2020). The cardiotoxic effects of acute and chronic grayanotoxin‐III in rats. Human & Experimental Toxicology, 39(3), 374–383. 31773988 10.1177/0960327119889668

[fsn34199-bib-0016] Eraslan, G. , Kanbur, M. , & Silici, S. (2009). Effect of carbaryl on some biochemical changes in rats: The ameliorative effect of bee pollen. Food and Chemical Toxicology, 47(1), 86–91. 18996165 10.1016/j.fct.2008.10.013

[fsn34199-bib-0017] Eraslan, G. , Kanbur, M. , Silici, S. , Altinordulu, S. , & Karabacak, M. (2008). Effects of cypermethrin on some biochemical changes in rats: The protective role of propolis. Experimental Animals, 57(5), 453–460. 18946182 10.1538/expanim.57.453

[fsn34199-bib-0018] Esrefoglu, M. , Iraz, M. , Ates, B. , & Gul, M. (2012). Melatonin and CAPE are able to prevent the liver from oxidative damage in rats: An ultrastructural and biochemical study. Ultrastructural Pathology, 36(3), 171–178. 22559044 10.3109/01913123.2011.647262

[fsn34199-bib-0019] Geng, H. , Lan, R. , Singha, P. K. , Gilchrist, A. , Weinreb, P. H. , Violette, S. M. , Weinberg, J. M. , Saikumar, P. , & Venkatachalam, M. A. (2012). Lysophosphatidic acid increases proximal tubule cell secretion of profibrotic cytokines PDGF‐B and CTGF through LPA2‐ and Gαq‐mediated rho and αvβ6 integrin‐dependent activation of TGF‐β. The American Journal of Pathology, 181(4), 1236–1249. 22885106 10.1016/j.ajpath.2012.06.035 PMC3463629

[fsn34199-bib-0020] Hotta, S. , Uchiyama, S. , & Ichihara, K. (2020). Brazilian red propolis extract enhances expression of antioxidant enzyme genes *in vitro* and *in vivo* . Bioscience, Biotechnology, and Biochemistry, 84(9), 1820–1830. 32490727 10.1080/09168451.2020.1773756

[fsn34199-bib-0021] Inandiklioglu, N. , Doganyigit, Z. , Okan, A. , Kaymak, E. , & Silici, S. (2021). Nephroprotective effect of apilarnil in lipopolysaccharide‐induced sepsis through TLR4/NF‐κB signaling pathway. Life Sciences, 284, 119875. 10.1016/j.lfs.2021.119875 34384831

[fsn34199-bib-0022] Kolankaya, D. , Selmanoğlu, G. , Sorkun, K. , & Salih, B. (2002). Protective effects of Turkish propolis on alcohol‐ induced serum lipid changes and liver injury in male rats. Food Chemistry, 78(2), 213–217.

[fsn34199-bib-0023] Korish, A. A. , & Arafa, M. M. (2011). Propolis derivatives inhibit the systemic inflammatory response and protect hepatic and neuronal cells in acute septic shock. The Brazilian Journal of Infectious Diseases, 15(4), 332–338. 21861003

[fsn34199-bib-0024] Latchoumycandane, C. , Nagy, L. E. , & McIntyre, T. M. (2014). Chronic ethanol ingestion induces oxidative kidney injury through taurine‐inhibitable inflammation. Free Radical Biology & Medicine, 69, 403–416. 24412858 10.1016/j.freeradbiomed.2014.01.001 PMC3960325

[fsn34199-bib-0025] Liu, B. C. , Tang, T. T. , Lv, L. L. , & Lan, H. Y. (2018). Renal tubule injury: A driving force toward chronic kidney disease. Kidney International, 93(3), 568–579. 29361307 10.1016/j.kint.2017.09.033

[fsn34199-bib-0026] Macias‐Perez, J. R. , Beltran‐Ramirez, O. , Vasquez‐Garzon, V. R. , Salcido‐Neyoy, M. E. , Martinez‐Soriano, P. A. , Ruiz‐Sanchez, M. B. , & Villa‐Trevino, S. (2013). The effect of caffeic acid phenethyl ester analogues in a modified resistant hepatocyte model. Anti‐Cancer Drugs, 24(4), 394–405. 23388162 10.1097/CAD.0b013e32835e9743

[fsn34199-bib-0027] Majiene, D. , Trumbeckaite, S. , Savickas, A. , & Toleikis, A. (2006). Influence of propolis water solution on heart mitochondrial function. The Journal of Pharmacy and Pharmacology, 58(5), 709–713. 16640841 10.1211/jpp.58.5.0017

[fsn34199-bib-0028] Meto, A. , Colombari, B. , Meto, A. , Boaretto, G. , Pinetti, D. , Marchetti, L. , Benvenuti, S. , Pellati, F. , & Blasi, E. (2020). Propolis affects *Pseudomonas aeruginosa* growth, biofilm formation, eDNA release and phenazine production: Potential involvement of polyphenols. Microorganisms, 8(2), 243. 32059431 10.3390/microorganisms8020243 PMC7074903

[fsn34199-bib-0049] Netíková, L. , Bogusch, P. , & Heneberg, P. (2013). Czech ethanol‐free propolis extract displays inhibitory activity against a broad spectrum of bacterial and fungal pathogens. Journal of Food Science, 78(9), M1421–M1429. 10.1111/1750-3841.12230 23915150

[fsn34199-bib-0029] Osman, I. H. , & Hafez Tantaway, A. A. (2013). Antioxidant activity and protective effects of commercial propolis on gentamicin induced nephrotoxicity in rabbits‐in vitro study. Turkish Journal of Biochemistry, 38(4), 409–415.

[fsn34199-bib-0030] Özcan, M. (2000). Use of propolis extract as a natural antioxidant for plant oils. Grasas y Aceites, 51(4), 251–253.

[fsn34199-bib-0031] Pasupuleti, V. R. , Sammugam, L. , Ramesh, N. , & Gan, S. H. (2017). Honey, propolis, and royal jelly: A comprehensive review of their biological actions and health benefits. Oxidative Medicine and Cellular Longevity, 2017, 1259510. 28814983 10.1155/2017/1259510 PMC5549483

[fsn34199-bib-0032] Pietta, P. G. , Gardana, C. , & Pietta, A. M. (2002). Analytical methods for quality control of propolis. Fitoterapia, 73(Suppl 1), S7–S20. 12495705 10.1016/s0367-326x(02)00186-7

[fsn34199-bib-0033] Pobiega, K. , Kraśniewska, K. , Derewiaka, D. , & Gniewosz, M. (2019). Comparison of the antimicrobial activity of propolis extracts obtained by means of various extraction methods. Journal of Food Science and Technology, 56(12), 5386–5395. 31749486 10.1007/s13197-019-04009-9 PMC6838241

[fsn34199-bib-0034] Pujirahayu, N. , Ritonga, H. , & Uslinawaty, Z. (2014). Properties and flavonoids content in propolis of some extraction method of raw propolis. International Journal of Pharmacy and Pharmaceutical Sciences, 6(6), 338–340.

[fsn34199-bib-0035] Rocha, B. A. , Bueno, P. C. , Vaz, M. M. , Nascimento, A. P. , Ferreira, N. U. , Moreno, G. , Rodrigues, M. R. , Costa‐Machado, A. R. , Barizon, E. A. , Campos, J. C. , de Oliveira, P. F. , Acésio, N. , Martins, S. , Tavares, D. C. , & Berretta, A. A. (2013). Evaluation of a propolis water extract using a reliable RP‐HPLC methodology and in vitro and in vivo efficacy and safety characterisation. Evidence‐based Complementary and Alternative Medicine, 2013, 670451. 23710228 10.1155/2013/670451 PMC3655582

[fsn34199-bib-0036] Saito, Y. , Tsuruma, K. , Ichihara, K. , Shimazawa, M. , & Hara, H. (2015). Brazilian green propolis water extract up‐regulates the early expression level of HO‐1 and accelerates Nrf2 after UVA irradiation. BMC Complementary and Alternative Medicine, 15(1), 421. 26611539 10.1186/s12906-015-0945-4 PMC4661975

[fsn34199-bib-0037] Sforcin, J. M. (2007). Propolis and the immune system: A review. Journal of Ethnopharmacology, 113(1), 1–14. 17580109 10.1016/j.jep.2007.05.012

[fsn34199-bib-0038] Sforcin, J. M. (2016). Biological properties and therapeutic applications of propolis. Phytotherapy Research, 30(6), 894–905. 26988443 10.1002/ptr.5605

[fsn34199-bib-0039] Sforcin, J. M. , & Bankova, V. (2011). Propolis: Is there a potential for the development of new drugs? Journal of Ethnopharmacology, 133(2), 253–260. 20970490 10.1016/j.jep.2010.10.032

[fsn34199-bib-0040] Silici, S. , & Baysa, M. (2020). Antioxidant, antiradical and antipyretic effects of olive oil extract of propolis. Journal of Apicultural Research, 59(5), 883–889.

[fsn34199-bib-0041] Speth, P. A. , Vree, T. B. , Neilen, N. F. , de Mulder, P. H. , Newell, D. R. , Gore, M. E. , & de Pauw, B. E. (1987). Propylene glycol pharmacokinetics and effects after intravenous infusion in humans. Therapeutic Drug Monitoring, 9(3), 255–258. 3672566 10.1097/00007691-198709000-00001

[fsn34199-bib-0042] Susanto, A. , Purwanto, B. , Mudigdo, A. , & Suroto, S. (2018). Nephro‐protective effect of the Indonesian propolis extract on unilateral renal ureter obstructive damage. Proceedings of the Mid‐International Conference on Public Health; Sebelas Maret University.

[fsn34199-bib-0043] Takemura, T. , Urushisaki, T. , Fukuoka, M. , Hosokawa‐Muto, J. , Hata, T. , Okuda, Y. , Hori, S. , Tazawa, S. , Araki, Y. , & Kuwata, K. (2012). 3,4‐Dicaffeoylquinic acid, a major constituent of Brazilian propolis, increases TRAIL expression and extends the lifetimes of mice infected with the influenza a virus. Evidence‐based Complementary and Alternative Medicine, 2012, 946867. 21876716 10.1155/2012/946867 PMC3163148

[fsn34199-bib-0044] Teles, F. , da Silva, T. M. , da Cruz Júnior, F. P. , Honorato, V. H. , de Oliveira, C. H. , Barbosa, A. P. F. , de Oliveira, S. G. , Porfírio, Z. , Libório, A. B. , Borges, R. L. , & Fanelli, C. (2015). Brazilian red propolis attenuates hypertension and renal damage in 5/6 renal ablation model. PLoS One, 10(1), 1–15. 10.1371/journal.pone.0116535 PMC4301812 25607548

[fsn34199-bib-0045] Tosi, B. , Donini, A. , Romagnoli, C. , & Bruni, A. (1996). Antimicrobial activity of some commercial extracts of propolis prepared with different solvents. Phytotherapy Research, 10(4), 335–336.

[fsn34199-bib-0046] van Kooten, C. , Daha, M. R. , & van Es, L. A. (1999). Tubular epithelial cells: A critical cell type in the regulation of renal inflammatory processes. Experimental Nephrology, 7(5–6), 429–437. 10559641 10.1159/000020622

[fsn34199-bib-0047] Yard, B. A. , Daha, M. R. , Kooymans‐Couthino, M. , Bruijn, J. A. , Paape, M. E. , Schrama, E. , van Es, L. A. , & van der Woude, F. J. (1992). IL‐1 alpha stimulated TNF alpha production by cultured human proximal tubular epithelial cells. Kidney International, 42(2), 383–389. 1405321 10.1038/ki.1992.299

[fsn34199-bib-0048] Ye, M. , Xu, M. , Ji, C. , Ji, J. , Ji, F. , Wei, W. , Yang, S. , & Zhou, B. (2019). Alterations in the transcriptional profile of the liver tissue and the therapeutic effects of propolis extracts in alcohol‐induced steatosis in rats. Anais da Academia Brasileira de Ciências, 91(3), e20180646. 31411259 10.1590/0001-3765201920180646

